# Limited impact of chytridiomycosis on juvenile frogs in a recovered species

**DOI:** 10.1007/s00442-023-05406-w

**Published:** 2023-06-22

**Authors:** Matthijs Hollanders, Laura F. Grogan, Hamish I. McCallum, Laura A. Brannelly, David A. Newell

**Affiliations:** 1 Faculty of Science and Engineering, Southern Cross University, Lismore, NSW Australia; 2 Centre for Planetary Health and Food Security, and School of Environment and Science, Griffith University, Southport, QLD Australia; 3 Veterinary BioSciences, Faculty of Veterinary and Agricultural Sciences, The University of Melbourne, Werribee, VIC Australia

**Keywords:** Amphibian, Chytridiomycosis, Life stage, *Batrachochytrium dendrobatidis*, Photographic mark-recapture, Multievent

## Abstract

The amphibian chytrid fungus *Batrachochytrium dendrobatidis* (*Bd*) has caused catastrophic frog declines on several continents, but disease outcome is mediated by a number of factors. Host life stage is an important consideration and many studies have highlighted the vulnerability of recently metamorphosed or juvenile frogs compared to adults. The majority of these studies have taken place in a laboratory setting, and there is a general paucity of longitudinal field studies investigating the influence of life stage on disease outcome. In this study, we assessed the effect of endemic *Bd* on juvenile *Mixophyes fleayi* (Fleay’s barred frog) in subtropical eastern Australian rainforest. Using photographic mark-recapture, we made 386 captures of 116 individuals and investigated the effect of *Bd* infection intensity on the apparent mortality rates of frogs using a multievent model correcting for infection state misclassification. We found that neither *Bd* infection status nor infection intensity predicted mortality in juvenile frogs, counter to the expectation that early life stages are more vulnerable to disease, despite average high infection prevalence (0.35, 95% HDPI [0.14, 0.52]). Additionally, we found that observed infection prevalence and intensity were somewhat lower for juveniles than adults. Our results indicate that in this *Bd*-recovered species, the realized impacts of chytridiomycosis on juveniles were apparently low, likely resulting in high recruitment contributing to population stability. We highlight the importance of investigating factors relating to disease outcome in a field setting and make recommendations for future studies.

## Introduction

Amphibians have declined around the world in part due to the global spread of the amphibian chytrid fungus (*Batrachochytrium dendrobatidis*, hereafter *Bd*), which caused the lethal disease chytridiomycosis in more than 500 species around the world (Scheele et al. 2019). Disease susceptibility is influenced by a number of factors, including host species (Scheele et al. 2019), *Bd* lineage (O’Hanlon et al. 2018), history of *Bd* exposure (Knapp et al. 2016; Waddle et al. 2019; Hollanders et al. 2023), environmental conditions (Kriger and Hero 2007), and host life stage (Sauer et al. 2020). Recently metamorphosed (juvenile) frogs are often reported to be more susceptible to chytridiomycosis than older life stages, potentially caused by restructuring of the immune system that occurs during metamorphosis (Rollins-Smith et al. 2011; Waddle et al. 2019; Sauer et al. 2020; Humphries et al. 2022).

Clinical experiments have found decreased survival after *Bd* exposure for juveniles compared to subadults and adults, especially just after metamorphosis (Rachowicz et al. 2006; Ortiz-Santaliestra et al. 2013; Abu Bakar et al. 2016; Brannelly et al. 2018; Waddle et al. 2019). Recently metamorphosed *Anaxyrus americanus* infected in the lab were three times more likely to die than 4-week-old juveniles (Ortiz-Santaliestra et al. 2013). In *Litoria aurea*, infection intensities and mortality were higher for subadults than adults, and again higher for juveniles than subadults (Abu Bakar et al. 2016). High mortality in *Rana onca* immediately following metamorphosis was suggestive of reduced immunocompetence at this life stage (Waddle et al. 2019). Opposite effects have also been found, where older frogs were found to be more susceptible to disease and carrying higher infection intensities (Bradley et al. 2019). Nevertheless, for all the merits of laboratory studies, there can be confounding effects (e.g., thermal mismatches, Sauer et al. 2020) that limit extrapolation to field conditions.

Few longitudinal field studies have investigated the effects of life stage on disease outcome. Although some cross-sectional studies have hinted at increased vulnerability for juveniles (Walker et al. 2010; Russell et al. 2010), these types of studies are generally unsuitable to assess outcomes of infection. One 5-year study on *Rana sierrae* and *R. muscosa* found higher *Bd* infection intensities for juveniles than adults, and mortality at metamorphosis—known to occur in challenge experiments (Rachowicz et al. 2006)—was hypothesized to explain the small population sizes at some sites. However, this study did not track juveniles through time. Another 7-year study on *Bombina variegata* found decreased survival probabilities for juveniles compared to adults, but with large uncertainty due to the paucity of juvenile recaptures (Spitzen-van der Sluijs et al. 2017). Detailed field studies to assess the effect of *Bd* on juvenile frogs are warranted to gain a more complete understanding of host-pathogen interactions in the field.

To investigate juvenile susceptibility to chytridiomycosis, we conducted a 3.5-month photographic mark-recapture study of recently metamorphosed *Mixophyes fleayi* (Fleay’s barred frog) at a rainforest site on the east coast of Australia. This endangered narrow-range endemic stream frog has demonstrated a strong recovery following population collapse associated with the *Bd* epidemic, and adult populations are currently stable with chytridiomycosis-related mortality largely confined to individuals with high *Bd* loads (Newell et al. 2013; Quick et al. 2015; Hollanders et al. 2023). First, we compared *Bd* infection patterns (infection status and pathogen loads) between juveniles and adults over the same time period. Then, we used a novel multievent model to investigate juvenile susceptibility to chytridiomycosis and to quantify infection dynamics (rates of gaining and clearing infections) in a post-metamorphic cohort.

## Materials and methods

### Field surveys

We conducted 14 weekly surveys (20 February–3 June 2020), each separated by 7 days, for juvenile *Mixophyes fleayi* on a 500 m road transect adjacent to Brindle Creek (within 100 m) in Border Ranges National Park, New South Wales, Australia. *Mixophyes fleayi* are large stream-associated frogs with Brindle Creek adult males weighing on average 34 g and females weighing 68 g (Hollanders et al. 2023). For more details on the study species and site, see Hollanders et al. (2023). We selected the transect because juvenile frogs were observed to congregate along the roadside from summer through autumn in greater numbers than found along the creek, suggesting juveniles dispersed to this habitat post-metamorphosis. Surveys commenced on dark and frogs were located by eyeshine using a headtorch, photographed dorsally *in situ*, and captured in a fresh plastic bag. Frogs were weighed to the 0.01 g using a digital scale (Homgeek CX-128) and snout-urostyle length (SUL) was derived from photographs (see below). We sampled for *Bd* using sterile rayon-tipped swabs (Medical Wire & Equipment MW100), applying five strokes for each hind foot, inner thigh, flank, and along the midventer, yielding 35 total strokes per frog. At the start of each survey, we used a Kestrel 3500 Weather Meter to measure humidity and air pressure, and temperature was recorded every 2 hours with a datalogger (HOBO MX2201) installed in the surrounding rainforest for the duration of the study. Surveys lasted 2–4 h depending on the number of frogs detected, and the entire transect was always subjected to the same survey intensity.

### Photographic identification

**Fig. 1:**
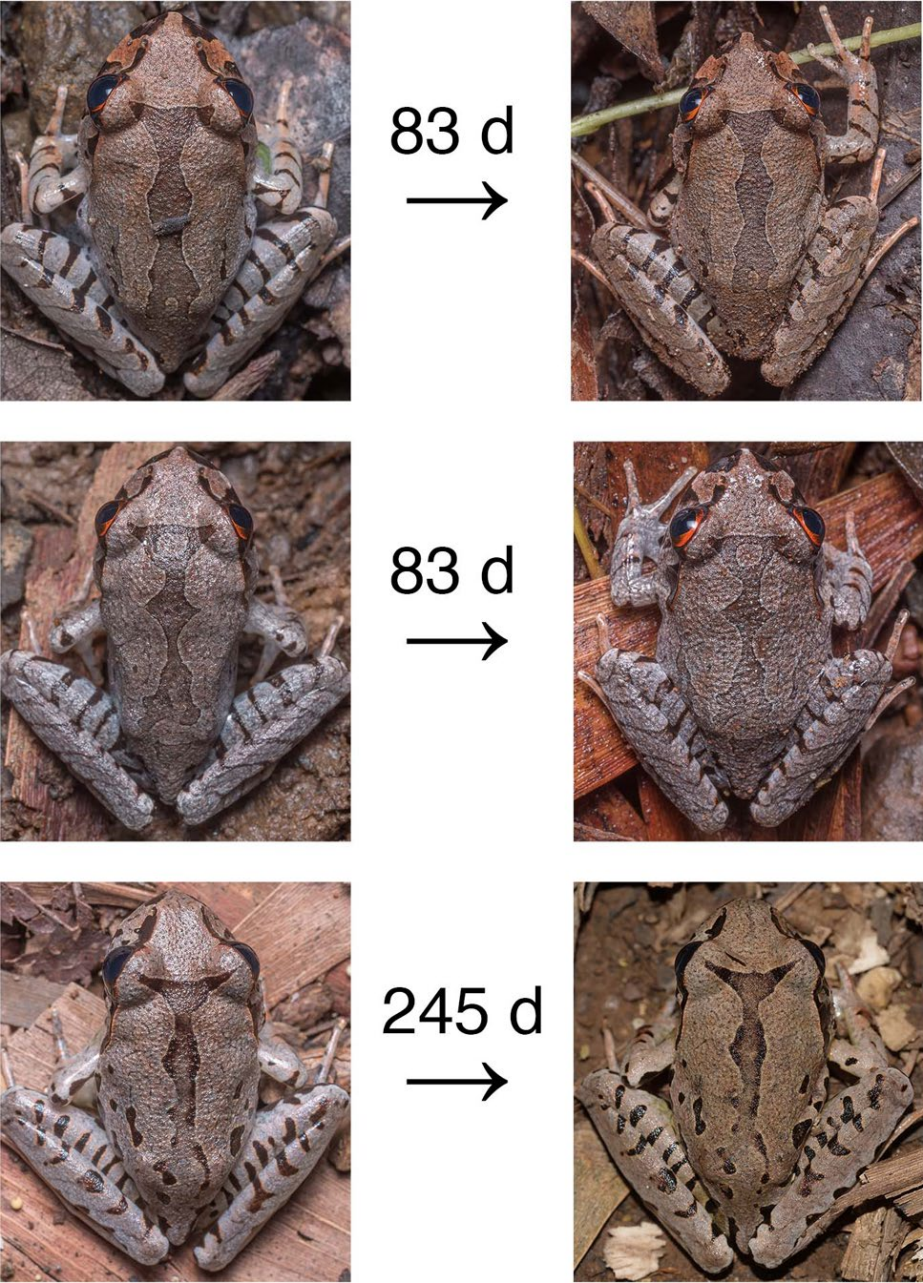
Dorsal patterns of three juvenile *Mixophyes fleayi*, with number of days between photographs, demonstrating pattern retention which facilitated individual identification. The individual with 245 days was recaptured after the field surveys described in this study

We used photographic mark-recapture because small body sizes impeded microchipping and because pattern retention facilitated individual identification (Fig. 1). Frogs were photographed dorsally to allow individual recognition during subsequent captures using a Nikon D750 DSLR, Sigma 105 mm macro lens, and diffused hotshoe flash (Yongnuo YN-560iii with Lumiquest III softbox). The focus ring was fixed in position for the duration of each survey to facilitate length measurements post-survey. After photographing a ruler at the start of each survey to calibrate measurements, pixel length was converted to the nearest 0.1 mm and SUL was measured using the Ruler Tool in Adobe Photograph CC 2018. Photos were matched to individuals manually by two independent investigators to limit errors, and equivocal identifications were discussed until a consensus was reached (Morrison et al. 2011).

### Detecting and quantifying *Bd* infections

We used Prepman^®^ Ultra (Applied Biosystems) to extract *Bd* DNA from swab samples and used qPCR to quantify infection intensities of swabs using synthetic ITS fragments as reference standards (Boyle et al. 2004; Hyatt et al. 2007). For details of the laboratory protocol, see Hollanders et al. (2023). Swab samples were run in duplicate and were considered positive when at least one well amplified > 1 ITS copy. Infection intensities are reported as log_10_ ITS gene copies per swab.

### Statistical analysis

#### *Bd* infection patterns and comparison with adults

To identify patterns in infection status and infection intensity, we fit logistic and linear regression models to the infection status and log_10_ infection intensities, respectively, of collected swab samples. In addition to the juveniles sampled in this study, we incorporated 92 swab samples collected from three mark-recapture surveys conducted for adult frogs at this site over the same study period (Hollanders et al. 2023). We included average temperature (from the datalogger) and (log) rainfall (extracted from interpolated data provided by the database SILO, Jeffrey et al. 2001) over the week prior to sample collection (and their interaction) as predictors on the probability of infection and the mean of infection intensities, respectively, and included random individual effects to account for repeated measures. The standard deviations (SDs) of the infection intensity distributions were estimated separately for each life stage.

#### Mark-recapture analysis

We fitted a multievent model to the mark-recapture data to investigate the effect of *Bd* infection on juvenile *M. fleayi* mortality and to assess infection dynamics (Hollanders and Royle 2022). This model incorporates state assignment errors (false-negative and false-positive errors) in both the swabbing and the qPCR protocols. We formulated the model with a continuous-time Arnason-Schwarz ecological process, with two alive states (uninfected and infected) and one dead state, with fortnightly primary occasion intervals (Schwarz et al. 1993; Glennie et al. 2022). To fit this model, robust design sampling is required (multiple “secondary” surveys within primary periods of assumed closure), so we pooled pairs of consecutive weeks into primary occasions, yielding eight primary occasions with two secondary surveys each (with two missing surveys). Although the closure assumption between consecutive weeks was likely violated, correct state assignment is notoriously low using swabs (30–60%, Shin et al. 2014; DiRenzo et al. 2018)—particularly using Prepman^®^ (Brannelly et al. 2020)—leading us to favor this model over a traditional Arnason-Schwarz model where the estimates for infection dynamics would be unreliable (Hollanders and Royle 2022). Like other mark-recapture models, we are modeling apparent mortality because true mortality is confounded with permanent emigration from the study area, which was likely to be common with dispersing frogs. However, comparing infection state-specific apparent mortality differences is still possible under the assumption that permanent emigration behavior is equal between states. Additionally, the Markov property of this model implies that state transitions are memoryless, so infection histories of individuals are not considered in the individual infection dynamics.

We modeled the parameters of the ecological process (hazard rates of mortality and gaining and clearing infections, log-link) and the probability of being infected with *Bd* at first capture (logit-link) as functions of body weight, body condition (scaled mass index, Peig and Green 2009), and average temperature over the primary occasion interval. We included *Bd* infection status and its interaction with body size as predictors on mortality to investigate whether more recently metamorphosed frogs were more vulnerable to infection. Rates of mortality and clearing infections also included latent time-varying individual *Bd* infection intensities as predictors. Recapture probabilities were modeled at the level of secondary surveys as logit-linear functions of temperature, humidity, and air pressure at the start of the survey, body weight, body condition, *Bd* infection status, *Bd* infection intensity, and random survey and individual effects. Note that body weight, body condition, and individual infection intensity are primary occasion-varying individual covariates. We modeled individual infection intensities (log_10_ *Bd* gene copies per swab) as coming from a Gaussian distribution with body weight, body condition, temperature, and random individual effects as predictors. We modeled the (true-positive) pathogen detection probabilities in the swabbing and qPCR processes as functions of individual and sample infection intensities, respectively, using Royle-Nichols models (e.g., $1-{\left(1-r\right)}^{n}$, Royle and Link 2006), and incorporated false-positive probabilities in both processes.

#### Variable selection

We used reversible jump Markov chain Monte Carlo (RJMCMC, Green 1995) for predictor variable selection and to test for the presence of false-positives in the swabbing and qPCR procedures. RJMCMC expands Metropolis-Hastings algorithms to sample from the posterior of a union of spaces of variable dimensions; i.e., it tests for whether the inclusion of certain parameters are consistent with the observed data. We applied RJMCMC to all predictor variables in both the infections model and the multievent model.

#### Model fitting

We used nimble 0.12.2 (de Valpine et al. 2017, 2022) in R 4.2.2 (R Core Team 2022) to sample from the joint posterior distributions using MCMC algorithms. All covariates including infection intensities were centered and scaled by two SDs (Gelman 2008). Given that this was a Bayesian analysis, prior distributions for all parameters were required. We used vague or weakly informative priors on most parameters: $\mathrm{Beta}\left(1,1\right)$ on back-transformed logit-linear intercepts, $\mathrm{Exponential}\left(1\right)$ on back-transformed log-linear intercepts of hazard rates, ${\text{t}}_{4}\left(3,1\right)$ on infection intensity intercepts, and ${\text{t}}_{4}^{\left(+\right)}\left(0,1\right)$ on coefficients and SDs of random effects. These prior distributions obey the constraints of the parameters (e.g., that probabilities are bounded between 0 and 1 and that rates and SDs are strictly positive) but remain otherwise largely uninformative leading to the information in the data dominating the posterior distributions. We used a $0.5$ prior probability on RJMCMC inclusion probabilities. We used more informative $\mathrm{Beta}\left(1,10\right)$ priors on false-positive probabilities in the pathogen detection protocol. Bounded (Beta, Exponential) prior distributions were transformed to the unbounded real line to improve MCMC performance using nimbleNoBounds (Pleydell 2022).

To account for missing values in covariate matrices, we imputed primary occasion-level body weight and body condition values for each primary occasion that an individual was not observed using submodels sampled by MCMC (Gelman et al. 2013). For body condition, missing values were mean-imputed with random individual effects. For body weight, we fitted a linear growth model with correlated random individual intercepts and slopes—we refrained from modeling growth asymptotically because linear growth seemed reasonable, with the largest frog weighing < 20% of an average adult male frog.

For both the infection patterns models and the multievent model, we ran four chains for 50,000 iterations after discarding 10,000 as burn-in and thinned each chain by 10, yielding 20,000 posterior draws. We summarized posterior distributions with medians and 95% highest posterior density intervals (HPDI) and report RJMCMC inclusion probabilities where applicable. We present intercepts on the original scale for ease of interpretation (e.g., probabilities, rates), coefficients as untransformed from the link function, and report the SDs of the normally distributed random effects (also on the scale of the link function). Coefficients and false-positives were summarized from the full posterior distribution, including iterations where they were excluded by RJMCMC and toggled to 0.

## Results

### Sampling summary

**Fig. 2:**
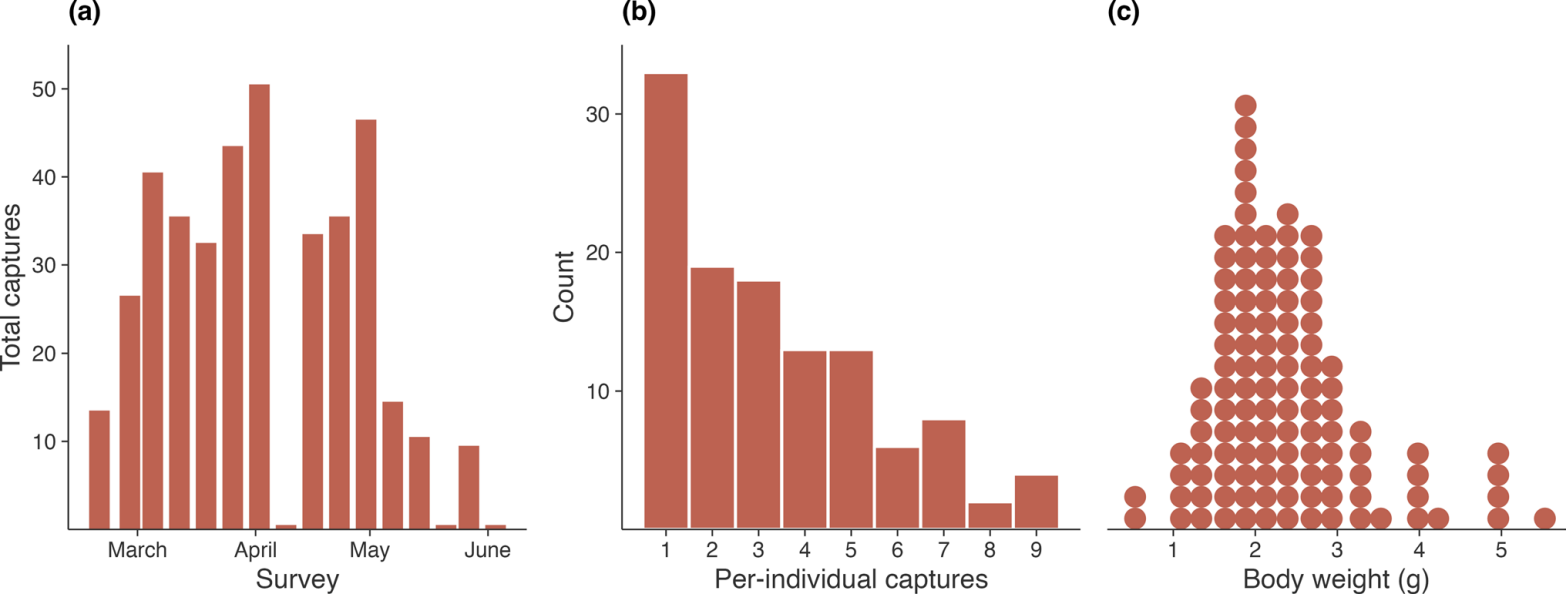
**a.** Number of unique individual frogs captured per survey (note two missing surveys in April and May). **b.** Histogram of number of captures per individual frog. **c.** Distribution of frog body weights. Each dot represents a frog, summarized by the mean of the measurements during the study

We made 386 captures of 116 unique juvenile *Mixophyes fleayi* (Fig. 2). The number of individuals captured per survey gradually increased, possibly as frogs metamorphosed and dispersed to the roadsides, peaking at 50 and gradually dropping to 0 after which surveys were terminated (Fig. 2a). Individuals were frequently recaptured, with 72% (83) being captured more than once, ranging from 1 to 9 captures per individual, with a median of three captures per individual (Fig. 2b). Measured body weights ranged from 0.46 to 6.41 g, corresponding to extremely recent metamorphs (i.e., within days) to young subadults (< 20% of adult males and < 10% of adult females) (Fig. 2c). Swab samples of 92 adults captured over the same period were included in the analyses.

### Bd infection patterns

**Fig. 3:**
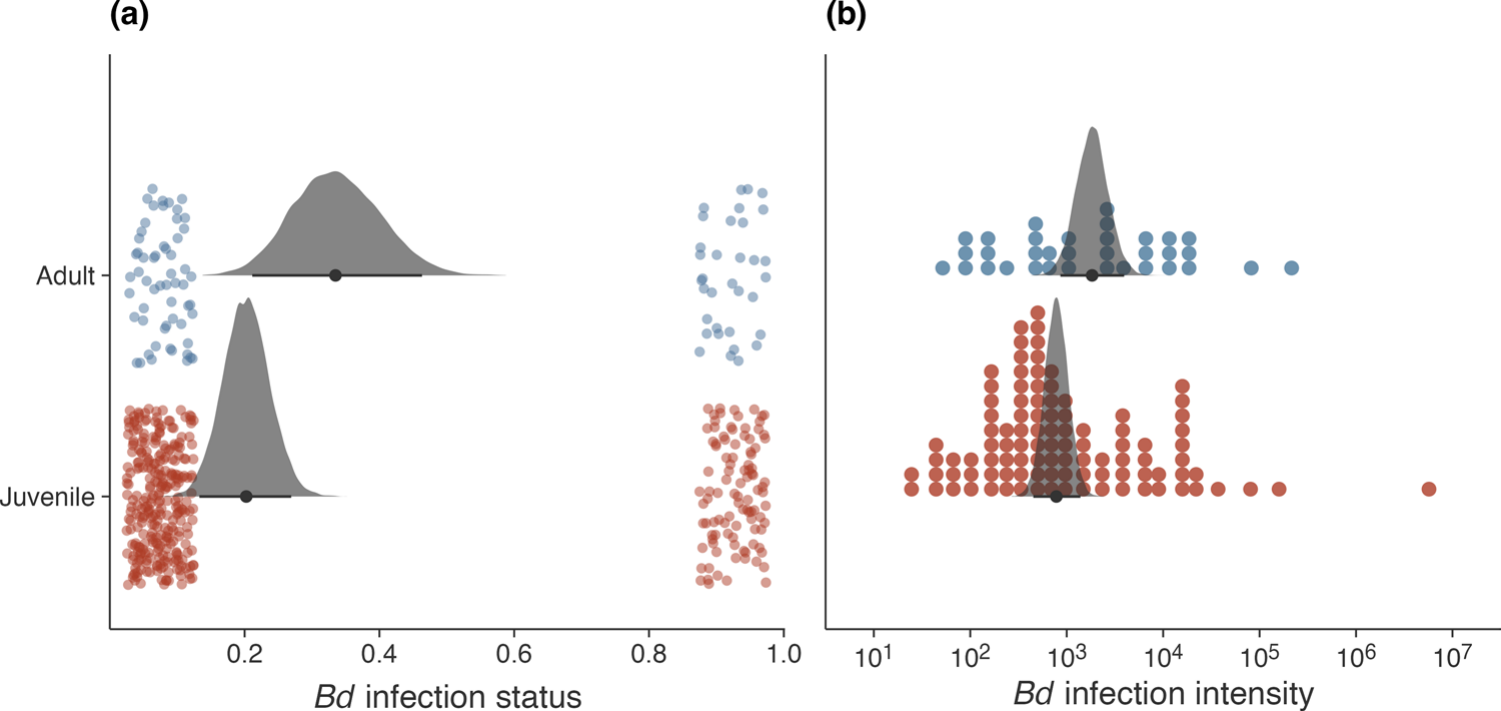
Infection patterns of *Bd* estimated from swab samples collected from adult and juvenile *Mixophyes fleayi*. **a.** Infection status (uninfected, left; infected, right) and posterior distributions of the probability of infection, which had an odds ratio of 1.98 [0.83, 3.71] for adults. **b.** Infection intensity (ITS gene copies per swab) and posterior distributions of the means of these distributions, with a difference of 12% [-2, 27]. Points represent individuals. Point intervals plotted under the posteriors are their medians and 95% HPDIs

Of 386 swabs collected from juvenile frogs, 101 (26%) had *Bd* detected and the average probability of infection (prevalence) was estimated to be 0.2 [0.13, 0.27]; for adults, we detected *Bd* on 35 out of 92 (38%) swabs, with an estimated probability of infection of 0.33 [0.21, 0.46] (Table 1, Fig. 3a). This difference was notable, with adults being 2 [0.88, 3.76] times more likely to return infected swabs. Mean estimated log_10_ infection intensities were 1.13 times [0.98, 1.28] higher for adults (3.26 [2.93, 3.6]) compared to juveniles (2.89 [2.65, 3.14]), with no major differences in the SDs of the distributions (Table 1, Figure 3b). We found some evidence for positive *Bd* infection status being associated with lower temperatures (− 0.22 [− 0.96, 0.01], 0.61 RJMCMC inclusion) and higher rainfall (0.65 [0, 1.18], 0.9 RJMCMC inclusion), but not for *Bd* intensity (Table 1). Note that these effects were not modeled separately for each life stage.

**Table 1:** Parameter estimates (median and 95% HDPI) and prior distributions of the logistic and linear Bd infection regression models summarized from 20,000 posterior draws

Function	Parameter	Median	95% HPDI	RJMCMC	Prior
*Bd* infection status					
	Intercept (juveniles)	0.2	[0.13, 0.27]		${\text{Beta}}\left(1,1\right)$
	Intercept (adults)	0.33	[0.21, 0.46]		${\text{Beta}}\left(1,1\right)$
	Temp	− 0.22	[− 0.96, 0.01]	0.61	${\text{t}}_{4}\left(0,1\right)$
	Rain	0.65	[0, 1.18]	0.9	${\text{t}}_{4}\left(0,1\right)$
	Temp $\times$ rain	0	[− 0.35, 0.82]	0.2	${\text{t}}_{4}\left(0,1\right)$
	*Individual effects (SD)*	1.19	[0.66, 1.73]		${\text{t}}_{4}^{+}\left(0,1\right)$
*Bd* infection intensity					
	Intercept (juveniles)	2.89	[2.65, 3.14]		${\text{t}}_{4}\left(3,1\right)$
	Intercept (juveniles)	3.26	[2.93, 3.6]		${\text{t}}_{4}\left(1,1\right)$
	Temp	0	[− 0.52, 0]	0.39	${\text{t}}_{4}\left(0,1\right)$
	Rain	0.03	[0, 0.54]	0.52	${\text{t}}_{4}\left(0,1\right)$
	Temp $\times$ rain	0	[− 0.05, 0.16]	0.07	${\text{t}}_{4}\left(0,1\right)$
	*Individual effects (SD)*	2	[1.52, 2.44]		${\text{t}}_{4}^{+}\left(0,1\right)$
	SD (juveniles)	0.64	[1.65, 2.19]		${\text{t}}_{4}^{+}\left(0,1\right)$
	SD (adults)	0.55	[1.23, 2.42]		${\text{t}}_{4}^{+}\left(0,1\right)$

All predictors were centered and scaled by two SDs. Random effects are italicized

### Mark-recapture analysis

**Fig. 4:**
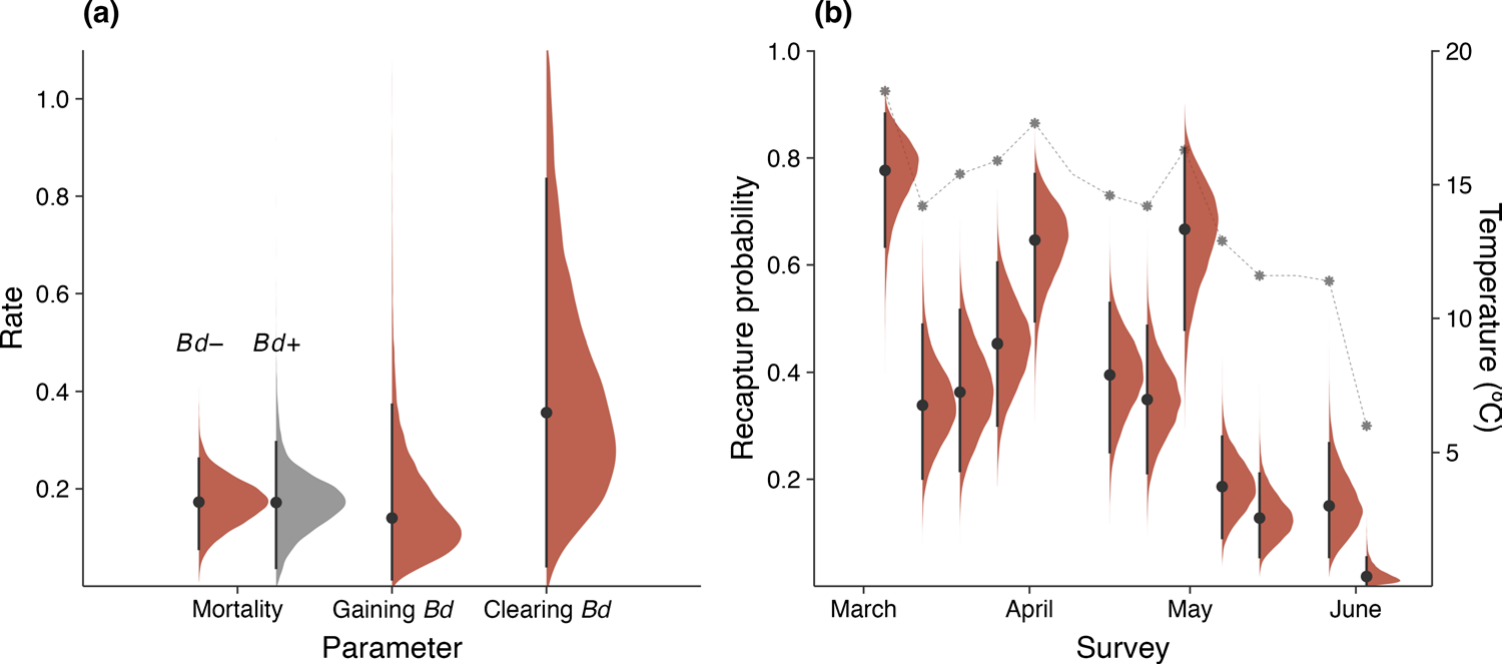
Posterior distributions (with medians and 95% HPDIs) of (**a**) fortnightly rates of apparent mortality and infection dynamics and (**b**) survey-specific predicted recapture probabilities. The mortality rate of infected individuals was derived as $\mathrm{exp}\left(\alpha +\beta \right)$, where $\alpha$ is the baseline log mortality hazard rate and $\beta$ is the effect of *Bd* infection status with average intensity. The grey stars in (**b**) show temperatures at the start of each survey

Average fortnightly apparent mortality rates of juvenile *M. fleayi* were 0.17 [0.07, 0.26], corresponding to a survival probability of 0.84 [0.77, 0.93], with no effects of *Bd* infection status or intensity on mortality (0.39 and 0.54 RJMCMC inclusion, respectively) (Table 2, Fig. 4a). Individual frogs were 2.63 [0.18, 8.1] times more likely to clear *Bd* infections (fortnightly hazard rate of 0.37 [0.03, 0.99], corresponding to a probability of 0.31 [0.05, 0.64]) than to gain infections (fortnightly hazard rate of 0.14 [0.01, 0.38], probability of 0.13 [0.01, 0.32]) (Fig. 4a). There were no clear effects of body weight, body condition, or temperature on mortality and infection dynamics (Table 2). Average recapture probabilities were 0.37 [0.23, 0.49] and strongly influenced by temperature (log odds change 2.44 [1.4, 3.6], 1 RJMCMC inclusion) (Fig. 4b). The probability of being infected with *Bd* at first capture was 0.42 [0.13, 0.7], and there was no support for effects of body weight, body condition, and temperature on this parameter (Table 2). This probability was similar to the average infection prevalence that was derived from monitoring the latent ecological states with MCMC (0.35 [0.14, 0.52]).

**Table 2:** Parameter estimates (median and 95% HDPI) and prior distributions of the multievent mark-recapture model summarized from 20,000 posterior draws

Function	Parameter	Median	95% HPDI	RJMCMC	Prior
Mortality ($\phi$)					
	Intercept	0.17	[0.07, 0.26]		${\text{Exp}}\left(1\right)$
	Body weight	0	[− 0.19, 1.09]	0.42	${\text{t}}_{4}\left(0,1\right)$
	Body weight $\times$ *Bd* status	0	[− 1.57, 0.84]	0.41	${\text{t}}_{4}\left(0,1\right)$
	Body condition	0	[− 0.55, 0.39]	0.26	${\text{t}}_{4}\left(0,1\right)$
	Temp (interval)	0	[− 1.03, 1.04]	0.37	${\text{t}}_{4}\left(0,1\right)$
	*Bd* status	0	[− 1.16, 1.01]	0.39	${\text{t}}_{4}\left(0,1\right)$
	*Bd* intensity	0	[− 2.76, 0.82]	0.54	${\text{t}}_{4}\left(0,1\right)$
Gaining *Bd* (${\psi }_{12}$)					
	Intercept	0.14	[0.01, 0.38]		${\text{Exp}}\left(1\right)$
	Body weight	0	[− 0.64, 2.28]	0.54	${\text{t}}_{4}\left(0,1\right)$
	Body condition	0	[− 1.7, 0.86]	0.43	${\text{t}}_{4}\left(0,1\right)$
	Temp (interval)	0	[− 1.34, 1.87]	0.47	${\text{t}}_{4}\left(0,1\right)$
Clearing *Bd* (${\psi }_{21}$)					
	Intercept	0.37	[0.03, 0.99]		${\text{Exp}}\left(1\right)$
	Body weight	− 1.08	[− 3.27, 0.25]	0.79	${\text{t}}_{4}\left(0,1\right)$
	Body condition	0	[− 0.76, 1.28]	0.4	${\text{t}}_{4}\left(0,1\right)$
	Temp (interval)	0	[− 1.91, 0.98]	0.46	${\text{t}}_{4}\left(0,1\right)$
	*Bd* intensity	0	[− 2.39, 0.8]	0.48	${\text{t}}_{4}\left(0,1\right)$
Recapture ($p$)					
	Intercept	0.37	[0.23, 0.49]		${\text{Beta}}\left(1,1\right)$
	Body weight	0	[− 0.17, 0.7]	0.3	${\text{t}}_{4}\left(0,1\right)$
	Body condition	0	[− 0.26, 0.67]	0.27	${\text{t}}_{4}\left(0,1\right)$
	Temp (survey)	2.44	[1.4, 3.6]	1	${\text{t}}_{4}\left(0,1\right)$
	Humidity (survey)	0	[− 0.46, 0.37]	0.23	${\text{t}}_{4}\left(0,1\right)$
	Air pressure (survey)	− 0.57	[− 1.32, 0]	0.74	${\text{t}}_{4}\left(0,1\right)$
	*Bd* status	0.69	[0, 1.84]	0.72	${\text{t}}_{4}\left(0,1\right)$
	*Bd* intensity	0	[− 0.74, 1.6]	0.46	${\text{t}}_{4}\left(0,1\right)$
	*Survey effects (SD)*	0.16	[0, 0.48]		${\text{t}}_{4}^{+}\left(0,1\right)$
	*Individual effects (SD)*	0.66	[0.01, 1.16]		${\text{t}}_{4}^{+}\left(0,1\right)$
First capture *Bd*+ ($\pi$)					
	Intercept	0.42	[0.13, 0.7]		${\text{Beta}}\left(1,1\right)$
	Body weight	0	[− 0.97, 1.17]	0.39	${\text{t}}_{4}\left(0,1\right)$
	Body condition	0	[− 0.23, 1.63]	0.52	${\text{t}}_{4}\left(0,1\right)$
	Temp (interval)	0	[− 1.03, 1.25]	0.41	${\text{t}}_{4}\left(0,1\right)$
*Bd* detection ($\delta ,\lambda$)					
	Swab true-positive (${r}_{\delta }$)	0.25	[0.15, 0.41]		${\text{Beta}}\left(1,1\right)$
	Swab false-positive (${\delta }_{21}$)	0	[0, 0.1]	0.52	${\text{Beta}}\left(1,10\right)$
	qPCR true-positive (${r}_{\lambda }$)	0.55	[0.45, 0.64]		${\text{Beta}}\left(1,1\right)$
	qPCR false-positive (${\lambda }_{21}$)	0.02	[0.01, 0.04]	0.99	${\text{Beta}}\left(1,10\right)$
Infection intensity ($\mu$)					
	Intercept	2.8	[2.48, 3.08]		${\text{t}}_{4}\left(3,1\right)$
	Body weight	0	[− 0.68, 0.05]	0.34	${\text{t}}_{4}\left(0,1\right)$
	Body condition	0	[− 0.29, 0.03]	0.17	${\text{t}}_{4}\left(0,1\right)$
	Temp (interval)	0	[− 0.59, 0.19]	0.25	${\text{t}}_{4}\left(0,1\right)$
	*Individual effects (SD)*	0.6	[0.23, 0.97]		${\text{t}}_{4}^{+}\left(0,1\right)$
	Population SD	0.41	[0.06, 0.69]		${\text{t}}_{4}^{+}\left(0,1\right)$
	Sampling process SD	0.37	[0.02, 0.64]		${\text{t}}_{4}^{+}\left(0,1\right)$
	Diagnostic process SD	0.5	[0.44, 0.58]		${\text{t}}_{4}^{+}\left(0,1\right)$

**Fig. 5:**
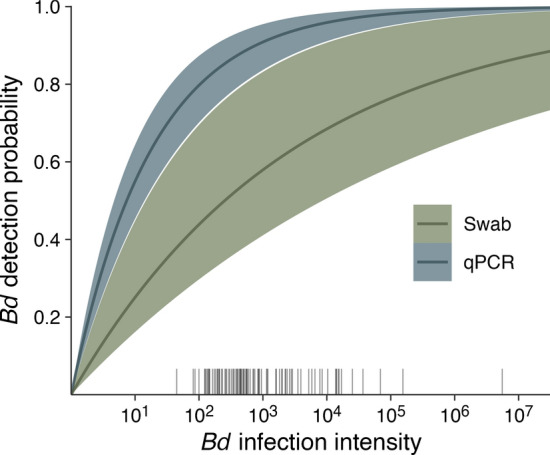
Prediction curves (medians and 95% equal-tailed intervals) of *Bd* detection probabilities in the swabbing and qPCR processes, estimated as $1-{\left(1-r\right)}^{n}$, where $r$ is the probability of detecting one log_10_ gene copies in each process and $n$ is the individual and sample infection intensity, respectively. The rug plot shows estimated time-varying individual infection intensities from captured individuals

The mean individual infection intensity estimated by the multievent model was 2.8 [2.48, 3.08] with an SD of 0.41 [0.06, 0.69]. Swab samples were estimated to have a (true-positive) probability of 0.25 [0.15, 0.41] to detect one log_10_ gene copies of *Bd*, corresponding to a probability of 0.55 [0.35, 0.78] to the detect the average infection (Fig. 5). There was limited evidence of false-positives in the swabbing process (0.52 RJMCMC inclusion), but the probability was estimated at 0.05 [0, 0.12] when included in the model. qPCR was estimated to have a probability of 0.55 [0.45, 0.64] to detect one log_10_ gene copies, yielding a probability of 0.89 [0.81, 0.95] to detect the average infection in each run (Fig. 5). There was strong support for the presence of false-positives in the qPCR procedure, albeit with low probability (0.02 [0.01, 0.04], 0.99 RJMCMC inclusion).

## Discussion

It has been widely assumed that juvenile frogs are more susceptible to chytridiomycosis than adult frogs, but our study suggests that this is not the case for Fleay’s barred frogs (*Mixophyes fleayi*). We found no evidence for the amphibian chytrid fungus *Bd* influencing mortality rates of a cohort of recently metamorphosed *M. fleayi*. Additionally, these juvenile frogs were nearly 3 times more likely to clear their *Bd* infections than to gain them. We estimated the odds of swab samples from juveniles being infected to be 0.5 [0.21, 0.92] times as high as for adults sampled concurrently with 0.89 [0.77, 1.01] times the mean infection intensities. True infection prevalence of juveniles (0.35 [0.14, 0.52]) was much higher, however, after correcting for imperfect pathogen detection in the multievent model fitted to the mark-recapture data.

We did not detect an effect of *Bd* infection (neither infection status nor intensity) on apparent mortality of juvenile frogs, and individuals cleared infections at higher rates than they gained them (Fig. 4a). By comparison, high infection intensities were associated with increased mortality in adult frogs at the same site (Hollanders et al. 2023). These results suggest that in this recovered species, the realized impact of chytridiomycosis is not higher for the juvenile life stage than for adults. It is possible that our sample included survivorship bias, where only the survivors of metamorphosis and early post-metamorphosis were included in the study. Previous studies have found increased mortality shortly after metamorphosis (Ortiz-Santaliestra et al. 2013; Abu Bakar et al. 2016; Waddle et al. 2019), which may have occurred with *M. fleayi* prior to inclusion in this study. However, even though some extremely recently metamorphosed individuals were included in the study, we found no evidence for age (using body weight as a proxy) influencing mortality. Laboratory challenge experiments are likely the only feasible way to estimate intrinsic susceptibility to chytridiomycosis during metamorphosis, but such studies are lacking in this species. Our results highlight that in the field, juveniles display limited susceptibility to chytridiomycosis after metamorphosis.

We found that adult *M. fleayi* were more likely to be infected with *Bd* and with slightly higher infection intensities than juveniles (Figure 3). The estimated 1.13 times higher infection intensities may simply reflect the larger surface areas swabbed on adult frogs. Although adult prevalence was estimated to be 0.33 [0.21, 0.46] from swabs over the study period, this is considerably higher than the average adult prevalence estimated over four years at this site (0.14 [0.09, 0.18]) (Hollanders et al. 2023). The higher prevalence over this particular time period may be due to the declining temperatures associated with autumn. With just a small sub-sample size of adults (n = 92) with only 35 swabs testing positive, our comparison is not decisive in making life stage comparisons. However, lower juvenile prevalence has been reported for another chytrid-affected species (*Litoria verreauxii alpina*) where it was suggested to facilitate demographic compensation where increased recruitment offsets decreased survival in adults due to chytrid impact (Scheele et al. 2015). The low infection intensities across life stages in *M. fleayi* may suggest a competent immune response to *Bd*, where the syntopic *Litoria pearsoniana* carried approximately 30% higher loads on average (4.01 [3.64, 4.4] log_10_ gene copies per swab) (Hollanders et al. 2023). Limited *Bd* impact on juvenile *M. fleayi* likely promotes recruitment, which was reported to increase in adult populations during an 8-year study in the early 2000s (Newell et al. 2013). This likely contributed to the large and stable populations observed today (Quick et al. 2015; Hollanders et al. 2023).

We are hesitant to suggest that juvenile frogs were less affected by *Bd* than adult frogs; however, our data suggest that juvenile *M. fleayi* are not experiencing greater disease impact in the field, contrary to the results of many previous studies (Sauer et al. 2020; Humphries et al. 2022) but somewhat in line with some recent results (Bradley et al. 2019). One study found *Bd*-related mortality in *Rana aurora* and *Pseudacris regilla* increased with age, but this study did not incorporate recently metamorphosed (< 4 weeks post-metamorphosis) (Bradley et al. 2019). Although intrinsic *Bd* susceptibility is difficult to assess in the field due to confounding effects (e.g., no exposure history and dosage), our results indicate that *Bd* is not an imminent threat for juvenile frogs in the field. A recent meta-analysis found that juveniles in laboratory challenge studies are often exposed to high *Bd* loads (1000× the amount required to find an effect on mortality), perhaps suggesting that laboratory studies have simulated unrealistic scenarios for populations where *Bd* is now endemic (Sauer et al. 2020).

Our results highlight the need to use recently developed statistical models that account for imperfect pathogen detection (DiRenzo et al. 2018, 2019; Hollanders and Royle 2022). Swabs were particularly unreliable, likely due to the Prepman^®^ DNA extraction protocol (Brannelly et al. 2020), with an estimated probability of 0.55 [0.35, 0.78] of detecting the average infection intensity on an individual (Fig. 5). As has been previously demonstrated, failing to account for state uncertainty in multistate mark-recapture models inflates the rates of infection dynamics but underestimates infection prevalence (Hollanders and Royle 2022). In our study, the odds of being infected derived from the multievent model were nearly two times higher than estimated using the swab samples alone as a proxy for infection. Accurate quantification of infection prevalence and dynamics requires accounting for misclassification errors.

Juvenile *M. fleayi* had high recapture probabilities, with an average of 0.37 [0.23, 0.49]—but going as high as 0.78 [0.63, 0.89]—and 83 individuals (72%) getting captured more than once over 14 surveys. By comparison, the only other (to our knowledge) longitudinal study on juvenile frogs did not report recapture probabilities but recaptured just 17 individuals (19%) over 19 surveys (median = 1 capture per individual) (Spitzen-van der Sluijs et al. 2017). Our results highlight the feasibility of future field studies on juvenile frogs, for which we recommend identifying sites where individuals congregate after dispersal from the breeding sites. Additionally, we stress the importance of identifying environmental covariates associated with activity patterns of the target species; in the case of both juvenile and adult *Mixophyes fleayi*, which live in subtropical rainforests, temperature was by far the most important driver (Hollanders et al. 2023).

Our results suggest that juvenile *M. fleayi* incur limited costs associated with *Bd* after post-metamorphic dispersal. Our study represents an important contribution to understanding the response of different life stages to a pathogen in host populations that have demonstrated recovery after initial epidemics. Limited impact of *Bd* likely results in high survivorship of juveniles and recruitment into adult populations, likely contributing to population stability observed across multiple sites. To our knowledge, this study is one of the first to explicitly investigate chytridiomycosis in juvenile frogs in a field setting and to compare mortality and infection dynamics with adults, especially in the context of high recapture rates which are essential to inference in mark-recapture analyses. We highlight the feasibility of future field studies on juvenile frogs to further investigate the effect of life stage on vulnerability to *Bd*.

## Data Availability

All data and code for analysis are available on https://github.com/mhollanders/mfleayi-juveniles
